# Exploring the age-dependent burden of periodontal disease in women across different socio-demographic levels

**DOI:** 10.1186/s41182-025-00886-3

**Published:** 2025-12-26

**Authors:** Min Wu, Man Liu, Yating Cao, Chanjuan Ye, Qian Qu, Yuanyuan Zheng, Shaowu Chen

**Affiliations:** 1 Department of Stomatology, Shenzhen Maternity and Child Healthcare Hospital, Southern Medical University, Shenzhen, Guangdong Province, China; 2 Department of Preventive Dentistry, School and Hospital of Stomatology, Fujian Medical University, Fuzhou, China; 3 School of Medical Technology and Nursing, Shenzhen Polytechnic University, Shenzhen, China

**Keywords:** Periodontal disease, Females, Specific physiological stages, Global burden of disease, Disability-adjusted life years, Socio-demographic index

## Abstract

**Background:**

Periodontal disease substantially affects women’s quality of life and shows sex-specific patterns due to physiological characteristics such as hormonal fluctuations, pregnancy, and menopause. However, most global assessments have focused on the general population and have not systematically characterized age specific burdens among women across different socio-demographic settings. This study addresses this gap by providing a life course analysis of the global, regional, and national incidence of periodontal disease in females from 1990 to 2021.

**Methods:**

Within the Global Burden of Disease (GBD) framework, we estimated female incidence and age-standardized rates (ASR) of periodontal disease across the life course in 204 countries and territories. Socio-Demographic Index (SDI)—incorporating per-capita income, years of schooling, and fertility in women under 25—was used to stratify locations into five levels (low, low-middle, middle, high-middle, high). We examined incidence trends and burden in eight specific physiological stages (childhood, adolescent, reproductive-age, prime reproductive-age, adult, perimenopausal, menopausal and older-age), and assessed the effects of oral health resources, diagnostic rates, and hormonal fluctuations. Our study presented point estimates with 95% confidence intervals (CIs). It evaluated the changing trends in the burden of Periodontal Disease using the estimated annual percentage change (EAPC) and percentage change.

**Results:**

Globally, the ASR differed across stages and was generally higher from the optimal reproductive age through older age. From 1990 to 2021, ASR rose steadily in childhood and adolescence, with a notable increase during the reproductive age in 2010–2014 (APC = 2.14). The optimal reproductive age showed increases in 1998–2005 and 2010–2014, adulthood and perimenopause exhibited fluctuating upward trends, while menopause and older age displayed divergent patterns. ASR–SDI associations were stage-dependent: negative correlations in childhood and adolescence, no significant association in adulthood and reproductive age, positive correlations in mid- to late-life, and SDI threshold effects (around SDI = 0.5, ASR tends to decrease; around SDI = 0.8, decelerated ASR growth).

**Conclusions:**

Policymakers should tailor public health strategies to high-burden regions and key life stages (e.g., reproductive and mid-to-late life), expand oral-health investment for children and adolescents in low-SDI areas, and strengthen screening/interventions for mid-older women in high-SDI regions.

**Supplementary Information:**

The online version contains supplementary material available at 10.1186/s41182-025-00886-3.

## Introduction

Periodontal disease is a highly prevalent yet often overlooked chronic inflammatory condition encompassing gingivitis—confined to the gingiva and reversible with good oral hygiene—and periodontitis, in which inflammation spreads, leading to tissue destruction and alveolar bone resorption [1]. It impairs multiple aspects of quality of life, including psychological well-being, social interactions, and food choices [2]. Periodontal disease is the sixth most common disease globally, affecting about 10% of adults [3, 4]. Clinical manifestations vary with age, sex, lesion number and distribution, progression, biofilm load, and tooth position within the arch [5]. Although men generally exhibit a higher prevalence of periodontitis globally, women differ markedly from men because of unique physiological factors such as hormonal fluctuations, pregnancy, and menopause. Therefore, this study focuses on the female population to elucidate these sex-specific risk profiles. Thus, physiological changes in female sex hormones, the distribution of estrogen and progesterone receptors, and hormonal metabolism are central to understanding pathogenesis in women [6]. Numerous studies report associations between periodontitis and hormone-related events—menstruation, pregnancy, and menopause [7–10]. For example, gingival inflammation can worsen in ovulatory and premenstrual phases versus during menstruation [7]. Pregnancy increases periodontal disease prevalence with poorer quality of life, more systemic comorbidities, and adverse pregnancy outcomes [8]. And, in postmenopausal women, reduced circulating estrogen and progesterone exacerbate oral symptoms (pain, burning, dryness), favor bone loss in individuals with osteoporosis, which is associated with an increased risk of periodontitis progressions [9]. It is important to note that current evidence supports a correlation between osteoporosis and periodontitis, but a direct causal relationship has not been conclusively established. These physiological factors modulate periodontal inflammatory responses to plaque, heightening risk of onset and progression.

However, current evidence is limited and lacks generalizability. Many countries lack high-quality oral epidemiological surveys disaggregated by sex and age, with research often concentrating on a single life stage, such as pregnancy [8, 10] or menopause [9]. Meanwhile, prior Global Burden of Disease (GBD) summaries have mostly focused on overall populations [4, 11, 12], giving insufficient attention to the burden among female subgroups, socioeconomic determinants, and intervention effects—key issues in research on periodontal disease in women.

In this study, we first quantified the prevalence, incidence, and disability-adjusted life years (DALYs) of periodontal disease among females globally and by region from 1990 to 2021, together with trends. We compared differences across Socio-Demographic Index (SDI) regions and countries, identifying high-burden and rapidly increasing areas. Finally, we explored burden amplification related to special physiological stages such as pregnancy and menopause, providing evidence for gender-sensitive public health strategies. This study seeks to fill the gap in systematic evaluation of the global burden of periodontal disease in women, advancing interdisciplinary research between dentistry and maternal-child health. These data provide strong evidence for interventions related to SDG 3.8 (oral health coverage) and SDG Target 3.1 (reducing maternal mortality), and should guide maternal and child health institutions to incorporate periodontal screening into preconception and prenatal care packages to mitigate maternal–infant complications.

The GBD study is an important resource for understanding epidemiology—including the prevalence and incidence of disease, mortality, and disability-adjusted life years (DALYs). Compared with earlier disease studies using GBD data [4, 12], we obtained disease-specific information from the latest GBD 2021. Unlike previous periodontal disease research focusing on the general population [11, 13], the present study focuses on female subgroups and is more targeted. In addition, this study provides a more comprehensive categorization of the prevalence and incidence of disease, mortality, and risk factors by age, sex, geographic region, and SDI, with emphasis on SDI distributions and temporal trends of disease burden. The analysis is intended to assist clinicians, epidemiologists, and health policymakers in strengthening resource allocation and developing more robust public health strategies.

## Method

### Study population and data sources

This study used data from the GBD 2021 project to analyze the burden of periodontal disease among females worldwide from 1990 to 2021. GBD 2021 is supported by numerous collaborators who provide, review, and analyze large volumes of data to systematically assess global health and disease burden. Data sources include various epidemiological surveys, population registries, and national health statistics reports [13]. In GBD 2021, periodontal disease is defined as Community Periodontal Index of Treatment Needs (CPITN) grade IV, attachment loss (AL) > 6 mm, or probing depth (PD) > 5 mm, corresponding to GBD code u145 and ICD-10 codes K05 (periodontitis, gingivitis) and K06.9 (unspecified periodontal disease) [14]. Using the GBD Results Tool (https://vizhub.healthdata.org/gbd-results/), we extracted data on incidence and other metrics for periodontal disease in women from GBD 2021. Data were modeled using the Bayesian meta-regression tool DisMod-MR 2.1, with consistency and reliability assessed using the MR-BRT Bayesian meta-regression tool to ensure accurate incidence estimates [15].

The study population comprised females in 204 countries and territories. This analysis exclusively focused on female data; male data were not included in this specific study. SDI is strongly correlated with health indicators and comprehensively reflects development level [16]; it includes lag-distributed income per capita, mean years of schooling, and fertility rate among females under 25 at a given location [17]. According to the GBD 2021 report, the final SDI values must be multiplied by 100 for conversion, and countries/territories are presented by five-level SDI stratification [14] into low, low-middle, middle, high-middle, and high groups [17], to analyze burden differences across development levels [18] (Supplementary Figure S1). In addition, the analytic units of this study were age-, sex-, year-, and location-specific estimates rather than individual participants. GBD 2021 synthesizes multiple data sources to generate model-based incidence estimates for the entire female population in each country and year; therefore, a single sample size (n) is not directly available. Instead, we used the age-specific population denominators provided by GBD 2021.

### The female life course

Based on the Global Burden of Disease (GBD) framework [19, 20] and female-specific physiological characteristics (e.g., hormonal fluctuations, reproductive cycle, and menopause), we stratified the female life course into eight mutually exclusive stages. The classification rationale and specific definitions are as follows, ensuring consistency with the abstract and overall study logic:

Childhood (1–9 years): Defined per GBD age stratification standards [19], further subdivided into early childhood (1–4 years) and late childhood (5–9 years), corresponding to the pre-pubertal period with stable hormonal levels.

Adolescence (10–19 years): Demarcated by the onset of puberty and reproductive development, subdivided into early adolescence (10–14 years) and late adolescence (15–19 years) [20]; this stage aligns with the initiation of hormonal changes (e.g., estrogen rise) relevant to periodontal health.

Reproductive age (15–49 years): Adopting the World Health Organization (WHO) definition of childbearing age [20], spanning from sexual maturity (adolescence onset) to perimenopause, characterized by active reproductive function and significant hormonal fluctuations (e.g., pregnancy-related changes).

Prime reproductive age (20–39 years): Derived to resolve overlaps among adolescence, reproductive age, and perimenopause; this stage focuses on the period of optimal fertility (low maternal risk and high reproductive capacity) to avoid confounding in burden analysis.

Adulthood (20–59 years): Encompasses the full reproductive and pre-perimenopausal period, bridging prime reproductive age and perimenopause, with age boundaries based on GBD’s adult stratification [19] and female physiological transition (stable reproductive function to hormonal decline).

Perimenopause (40–59 years): Defined by the onset of menopausal symptoms (e.g., irregular menstrual cycles) to 1 year after the final menstrual period [20], with a ± 5-year buffer around the median menopause age to account for individual variability in hormonal decline.

Menopause (45–54 years): Centered on the typical age of natural menopause (global median ~ 49.5 years) [20], corresponding to the cessation of menstrual cycles and significant estrogen/progesterone reduction, a key physiological driver of periodontal risk.

Older age (≥ 60 years): Consistent with GBD’s definition of older adults [19], reflecting the post-menopausal period with stable (but low) hormonal levels and age-related health declines.

### Statistical analysis

#### Calculation of the age-standardized rates (ASR)

Age is an important determinant of disease burden. To compare disease burdens across regions and countries, and to accurately present differences in the burden of periodontal disease among females across locations and years, standardization of measures such as prevalence and incidence is required [21]. The ASR was estimated using the direct standardization method with the GBD 2021 world standard population as weights [22]. The ASR per 100,000 females was calculated as: ASR = $\frac{{\sum }_{i=1}^{N}{a}_{i}{W}_{i}}{{\sum }_{i=1}^{N}{W}_{i}}$, where ${a}_{i}$ denotes the age-specific incidence rate for age group *i*, ${W}_{i}$ is the number of individuals in the same age group from the GBD 2021 standard population statistics, and N is the total number of age groups.

### Estimated annual percentage change (EAPC) and joinpoint analysis

To investigate temporal trends in incidence of periodontal disease among women during 1990–2021, we used EAPC [23]. Assuming the natural logarithm of ASR follows a linear regression model: ln (ASR) = *α* + *β*x + ϵ, where *x* is year, *β* is the trend slope, *α* is the intercept, and *ϵ* is the error term. EAPC is calculated as: EAPC = 100 × (e^*β*^-1). When the 95% confidence interval (CI) of EAPC lies entirely above zero, the indicator shows an increasing trend; entirely below zero, a decreasing trend; if the 95% CI includes zero, the ASR change is not statistically significant [24]. For 1990–2021, we used segmented regression (Joinpoint regression) to identify key turning points [25], quantify annual percentage change (APC) and average annual percentage change (AAPC), and determine specific years of greatest change.

### Correlation between disease burden and SDI

We used geographically smoothed LOESS fitting with geom_smooth in ggplot2 to analyze correlations between female periodontal disease burden and SDI across 21 regions and 204 countries/territories [14]. We also applied Spearman correlation to assess associations between burden (e.g., ASR) and SDI [16], calculating correlation coefficients (r) and P values. All statistical analyses and figures were generated in R (version 4.2.2). Statistical significance was set at P < 0.05.

### Ethical statement

The data utilized in this study are publicly accessible from the GBD 2021 via the GBD Results Tool. As such, no additional ethical approval was required for this secondary analysis. The datasets were downloaded, stored securely on password-protected servers, and used solely for the purposes of this analysis. The data will be retained by the corresponding author and made available upon reasonable request to facilitate reproducibility of our findings.

## Results

### Global burden of periodontal disease in women across eight life stages

Building on the eight female life course stages defined by the GBD framework and female physiological characteristics—namely childhood, adolescence, reproductive age, prime reproductive age, adulthood, perimenopause, menopause, and older age (with clear age boundaries and physiological bases for each)—the following sections will systematically present the global burden characteristics of periodontal disease in women across these stages, including disease distribution, temporal trends, and regional heterogeneity.

**Fig. 1:**
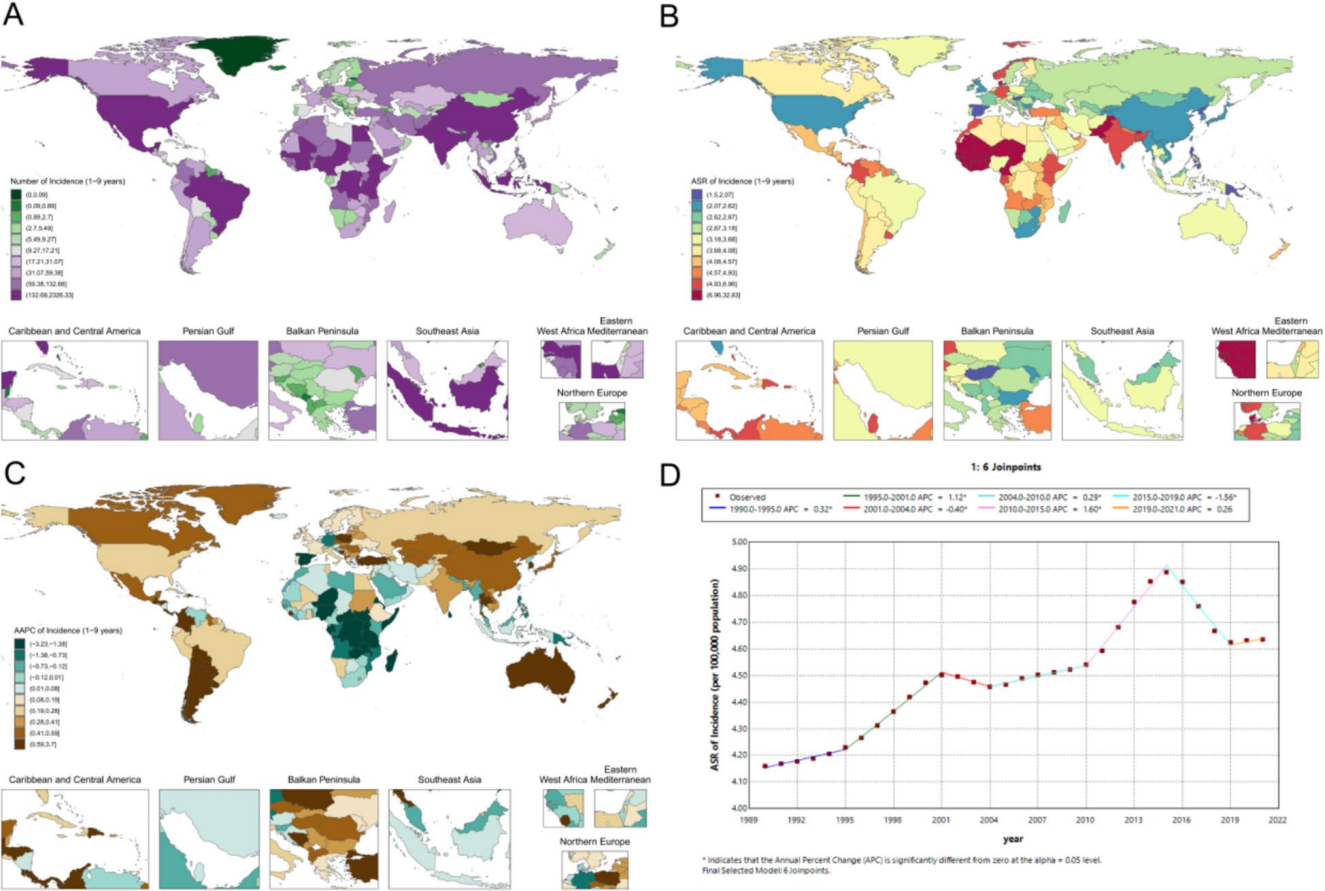
Childhood female periodontal disease metrics: **A** national distribution of cases in 2021; **B** national distribution of ASR in 2021; **C** national AAPC of ASR, 1990–2021; **D** global Joinpoint analysis of ASR, 1990–2021

In 2021, the estimated case counts and ASRs of childhood periodontal disease among females showed marked regional heterogeneity. Globally, there were 46.4 thousand cases (95% UI 41.6–51.1) (Fig. 1A), corresponding to an ASR of 4.64 (4.16–5.11) per 100,000 population (Fig. 1B). At the national level, substantial differences were observed: India had the highest ASR at 5.35 (2.22–10.33) per 100,000, followed by China at 2.31 (0.88–4.40) per 100,000, whereas high-income countries such as the United States, the United Kingdom, and Japan all had ASRs below 3.0 per 100,000 (Fig. 1A). In West Africa, the burden was also prominent, with Nigeria reporting an ASR of 3.31 (0.95–7.76) per 100,000, compared with 2.51 (0.86–5.61) per 100,000 in South Africa (Fig. 1A).

From 1990 to 2021, the global ASR increased modestly (AAPC 0.54 [0.31–0.76]) (Fig. 1D). A transient decline was observed during 2015–2019 (APC − 1.56, p < 0.05), followed by a slight rebound in 2021 (Fig. 1D). National trajectories varied: both China and India exhibited sustained increases, whereas Brazil and South Africa showed stable or slightly declining trends (Fig. 1C).

**Fig. 2:**
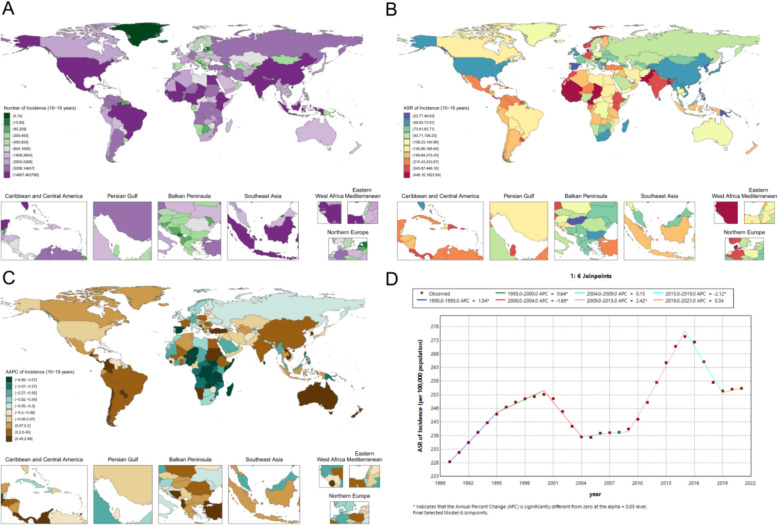
Adolescent female periodontal disease metrics: **A** national case distribution in 2021; **B** national ASR distribution in 2021; **C** national AAPC of ASR, 1990–2021; **D** global Joinpoint analysis, 1990–2021

In 2021, the global ASR of adolescent female periodontal disease was 72.1 (95% UI 39.6–126.8) per 100,000 population (Fig. 2B). Pronounced national gradients were observed, with Pakistan exhibiting the highest ASR at 820.2 (430.9–1377.8) per 100,000, followed by India at 351.6 (202.1–552.8) per 100,000. In contrast, estimates for China, the United States, and the United Kingdom were comparatively lower, at 72.1 (39.6–126.8), 72.8 (40.9–119.3), and 58.6 (30.4–104.6) per 100,000, respectively (Fig. 2B). West Africa also bore a considerable burden, with ASRs of 97.4 (42.6–192.9) per 100,000 in Nigeria and 66.6 (34.9–118.2) per 100,000 in South Africa (Fig. 2B).

From 1990 to 2021, the global ASR increased at an AAPC of 0.86% (0.37–1.35; p = 0.001) (Fig. 2D). National trends varied substantially: India showed the most rapid increase (AAPC 0.70% per year [0.65–0.75]; p < 0.001), China experienced a moderate rise (AAPC 0.48% per year [0.15–0.81]; p = 0.004), whereas West Africa demonstrated a declining trend (AAPC − 0.30% per year [− 0.38 to − 0.22]; p < 0.001) (Fig. 2C).

**Fig. 3:**
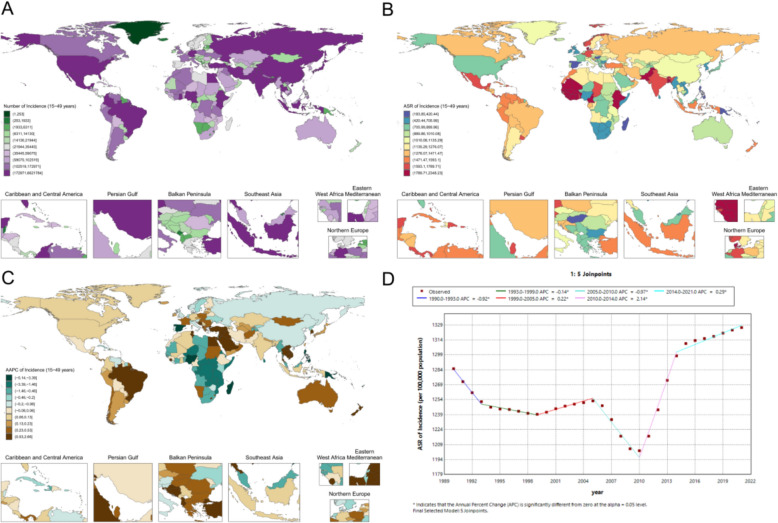
Reproductive-age female periodontal disease metrics: **A** national case distribution in 2021; **B** national ASR distribution in 2021; **C** national AAPC of ASR, 1990–2021; **D** global Joinpoint analysis, 1990–2021

In 2021, the global ASR of periodontal disease among women of reproductive age was 1192.3 (95% UI 684.7–1701.9) per 100,000 population, with pronounced regional heterogeneity (Fig. 3A, B). Marked national differences were observed, with particularly heavy burdens in West Africa: Sierra Leone had the highest ASR at 2348.2 (1526.1–3035.0) per 100,000, followed by the Gambia (2294.4 per 100,000) and Cape Verde (2255.6 per 100,000). By contrast, several Pacific Island countries exhibited relatively low ASRs, such as Kiribati at 183.9 (79.3–370.7) per 100,000. Among high-income countries, Denmark reached 2162.7 (1331.1–2916.9) per 100,000, exceeding estimates for the United States and the United Kingdom. China’s ASR was 1058.5 (648.0–1546.5) per 100,000, lower than that of India (1759.7 per 100,000) and Pakistan (2092.7 per 100,000).

From 1990 to 2021, a five-joinpoint model (Fig. 3D) indicated a significant increase during 2010–2014 (APC 2.14; p < 0.05), with fluctuating trends in other periods. Regional trajectories also differed (Fig. 3C), with Saudi Arabia showing an increasing trend (AAPC 1.82; p < 0.05), Croatia a decreasing trend (AAPC − 0.89; p < 0.05), and China (AAPC 0.53; p < 0.05) and India (AAPC 0.61; p < 0.05) both exhibiting moderate long-term increases.

**Fig. 4:**
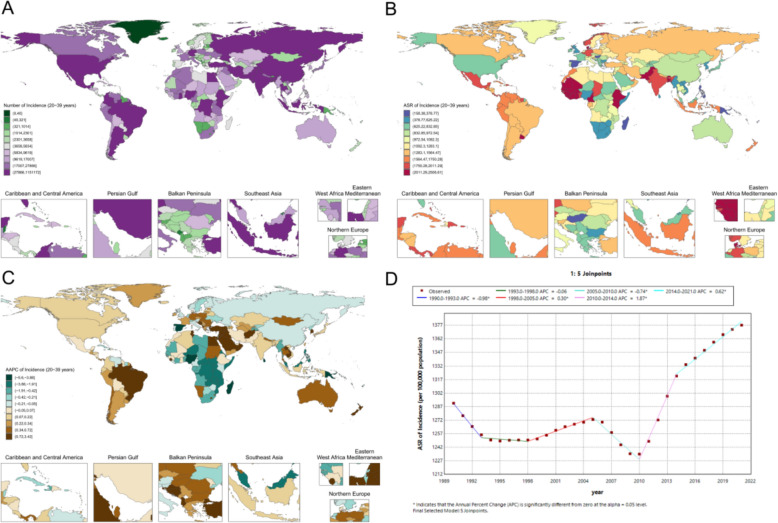
Prime reproductive-age female periodontal disease metrics: **A** national case distribution in 2021; **B** national ASR distribution in 2021; **C** national AAPC, 1990–2021; **D** global Joinpoint analysis, 1990–2021

In 2021, the global ASR of periodontal disease among women at prime reproductive age was 1287.5 (95% UI 726.3–1850.2) per 100,000 population (Fig. 4A, B). Substantial regional heterogeneity was observed, with particularly high burdens in South Asia and parts of West Africa: Pakistan (2369.8 [1487.0–3140.8] per 100,000), Bhutan (2239.3 [1253.0–3175.3] per 100,000), and India (2000.8 [1284.8–2723.7] per 100,000), while Sierra Leone and the Gambia both exceeded 2400 per 100,000. In contrast, several Pacific Island countries had relatively low ASRs, including Tokelau at 200.6 (88.3–404.0) per 100,000, with Nauru and Niue each below 230 per 100,000. Within Asia, the ASR among women of prime reproductive age was 936.5 (559.9–1448.4) per 100,000 in China, 422.6 (194.9–739.3) per 100,000 in the Democratic People’s Republic of Korea (DPRK), and 523.8 (238.6–1026.1) per 100,000 in South Korea. Among high-income countries, Denmark showed the highest burden at 2506.6 (1568.4–3253.6) per 100,000, followed by Norway at 2002.2 (1073.8–3002.5) and Switzerland at 1020.3 (475.9–1952.5) per 100,000 (Fig. 4A, B).

From 1990 to 2021, a five-joinpoint model (Fig. 4D) indicated significant increases during 1998–2005 (APC 0.30; p < 0.05), 2010–2014 (APC 1.87; p < 0.05), and 2014–2021 (APC 0.62; p < 0.05). Regional trends also varied (Fig. 4C): Cambodia exhibited a sustained increase (AAPC 0.87; p < 0.0001), Burkina Faso a slight but significant rise (AAPC 0.02; p = 0.0043), whereas China showed no significant long-term change (AAPC − 0.10; p = 0.4013) and India demonstrated a slow upward trend (AAPC 0.09; p < 0.0001).

**Fig. 5:**
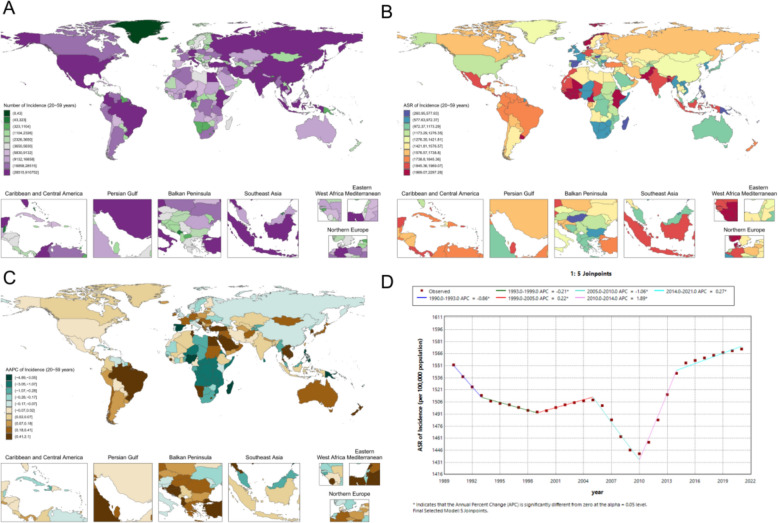
Adult female periodontal disease metrics: **A** national case distribution in 2021; **B** national ASR distribution in 2021; **C** national AAPC of ASR, 1990–2021; **D** global Joinpoint analysis, 1990–2021

In 2021, the global ASR of periodontal disease among adult women was 1468.2 (95% UI 826.5–2110.8) per 100,000 population (Fig. 5A, B). The burden was particularly high in South Asia, West Africa, and parts of the Caribbean: Bangladesh (2014.6 [1054.9–2922.3] per 100,000), Burkina Faso (2093.7 [1166.4–2879.1] per 100,000), and Cameroon (2123.0 [1227.6–2866.9] per 100,000), while Trinidad and Tobago and Panama each exceeded 1,900 per 100,000. In contrast, several Pacific Island states and parts of southern Africa showed relatively low ASRs, including the Cook Islands at 454.7 (192.6–890.1) per 100,000, Botswana at 588.7 (246.8–1162.5) per 100,000, and Zimbabwe at 626.1 (265.0–1,133.9) per 100,000 (Fig. 5A, B). In Asia, China’s ASR was 1395.1 (887.0–1969.0) per 100,000, whereas Japan and Singapore had lower estimates of 1064.0 (493.8–1848.6) and 980.8 (440.5–1778.7) per 100,000, respectively. Among high-income countries, Denmark exhibited one of the highest burdens at 2297.2 (1431.4–2973.7) per 100,000, compared with 1968.8 (1028.9–2909.5) per 100,000 in Germany and 1685.1 (839.3–2667.3) per 100,000 in Canada (Fig. 5A, B).

From 1990 to 2021, a five-joinpoint model (Fig. 5D) identified significant increases in 1998–2005 (APC 0.25; p < 0.05) and 2012–2018 (APC 0.41; p < 0.05). Regional trends also differed (Fig. 5C): Vietnam showed a sustained increase (AAPC 0.80; p < 0.0001), Libya a marked decline (AAPC − 0.42; p < 0.0001), while China exhibited no significant long-term change (AAPC − 0.09; p = 0.2600) and Pakistan demonstrated a slight decreasing trend (AAPC − 0.06; p < 0.0001).

**Fig. 6:**
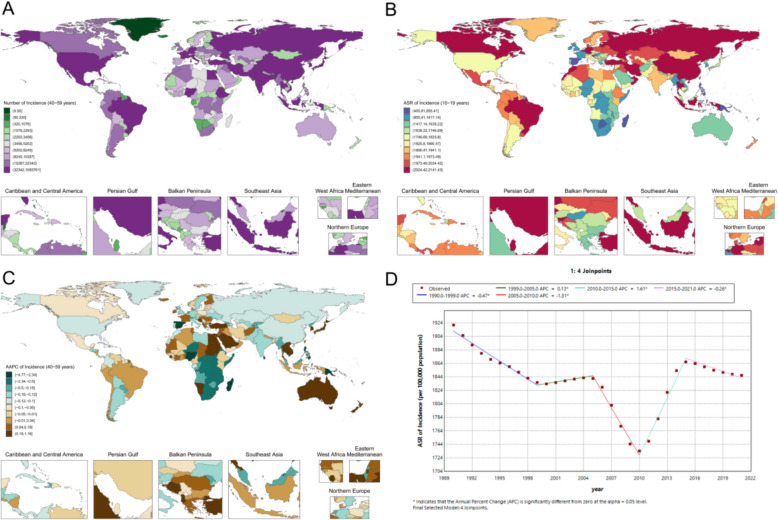
Perimenopausal female periodontal disease metrics: **A** national case distribution in 2021; **B** national ASR distribution in 2021; **C** national AAPC of ASR, 1990–2021; **D** global Joinpoint analysis, 1990–2021

In 2021, the global ASR of periodontal disease among perimenopausal women was 1582.6 (95% UI 910.4–2255.1) per 100,000 population (Fig. 6A, B). The burden was particularly elevated in parts of Southeast Asia, West Africa, and the Caribbean: Vietnam (1892.4 [1056.7–2728.2] per 100,000), Cambodia (1763.8 [989.5–2538.1] per 100,000), and Laos (1645.2 [908.4–2382.1] per 100,000), while the Gambia and Ghana each exceeded 1900 per 100,000 and Trinidad and Tobago and Barbados also showed high ASRs. By contrast, several Pacific Island states and countries in southern Africa exhibited relatively low rates, including the Cook Islands at 321.5 (142.8–500.3) per 100,000, Botswana at 487.2 (218.6–755.8) per 100,000, and Zimbabwe at 563.9 (250.7–877.1) per 100,000 (Fig. 6A, B). In East Asia, China’s ASR was 1642.8 (1025.3–2260.4) per 100,000, compared with 987.3 (450.6–1,524.1) per 100,000 in the Democratic People’s Republic of Korea and 923.5 (510.8–1336.2) per 100,000 in South Korea. Among high-income countries, Denmark had one of the highest burdens at 2145.7 (1320.9–2970.5) per 100,000, followed by Norway at 1892.3 (1010.6–2774.0) and Switzerland at 1287.6 (598.4–1976.8) per 100,000.

From 1990 to 2021, a five-joinpoint model (Fig. 6D) indicated significant increases during 2005–2011 (APC 0.21; p < 0.05) and 2016–2021 (APC 0.35; p < 0.05). Regional trends also varied (Fig. 6C): Cambodia showed a sustained increase (AAPC 0.45; p < 0.0001), Libya a significant decline (AAPC − 0.16; p < 0.0001), whereas China exhibited no significant long-term change (AAPC − 0.10; p = 0.1496) and Pakistan demonstrated a slight decreasing trend (AAPC − 0.12; p < 0.0001).

**Fig. 7:**
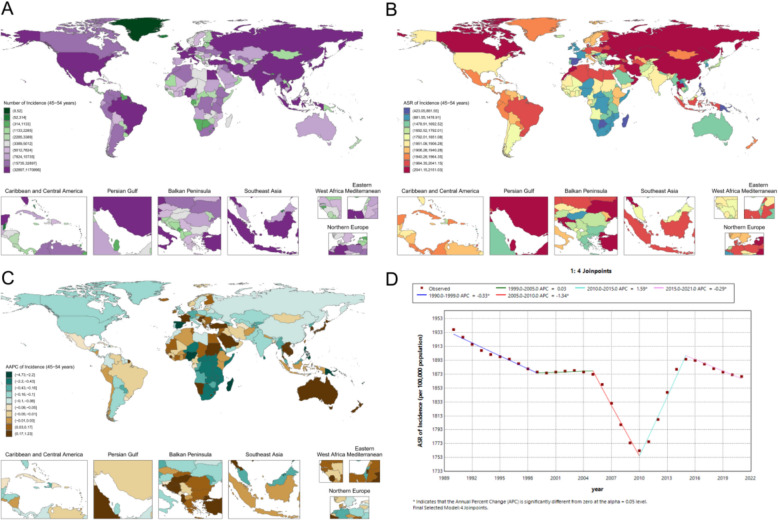
Menopausal female periodontal disease metrics: **A** national case distribution in 2021; **B** national ASR distribution in 2021; **C** national AAPC of ASR, 1990–2021; **D** global Joinpoint analysis, 1990–2021

In 2021, ASR of periodontal disease among postmenopausal women showed substantial national variation (Fig. 7A, B). The ASR was 2,089.3 (95% UI 1397.1–2694.7) per 100,000 population in mainland China, 2078.3 (1121.5–3005.8) per 100,000 in Taiwan, China, and 2036.9 (1166.6–2761.8) per 100,000 in Indonesia. By contrast, the ASR was relatively lower in the Democratic People’s Republic of Korea at 1552.6 (705.0–2574.2) per 100,000 and in Cambodia at 1791.3 (819.1–2773.7) per 100,000.

From 1990 to 2021, temporal trends diverged across countries (Fig. 7C, D): China showed no significant long-term change (AAPC − 0.0908; 95% CI − 0.2063 to 0.0248; p = 0.123556), whereas the DPRK experienced a significant decline (AAPC − 0.2177; 95% CI − 0.3206 to − 0.1148; p = 3.4e−05) and Cambodia a significant increase (AAPC 0.4203; 95% CI 0.3458–0.4948; p = 0.0).

**Fig. 8:**
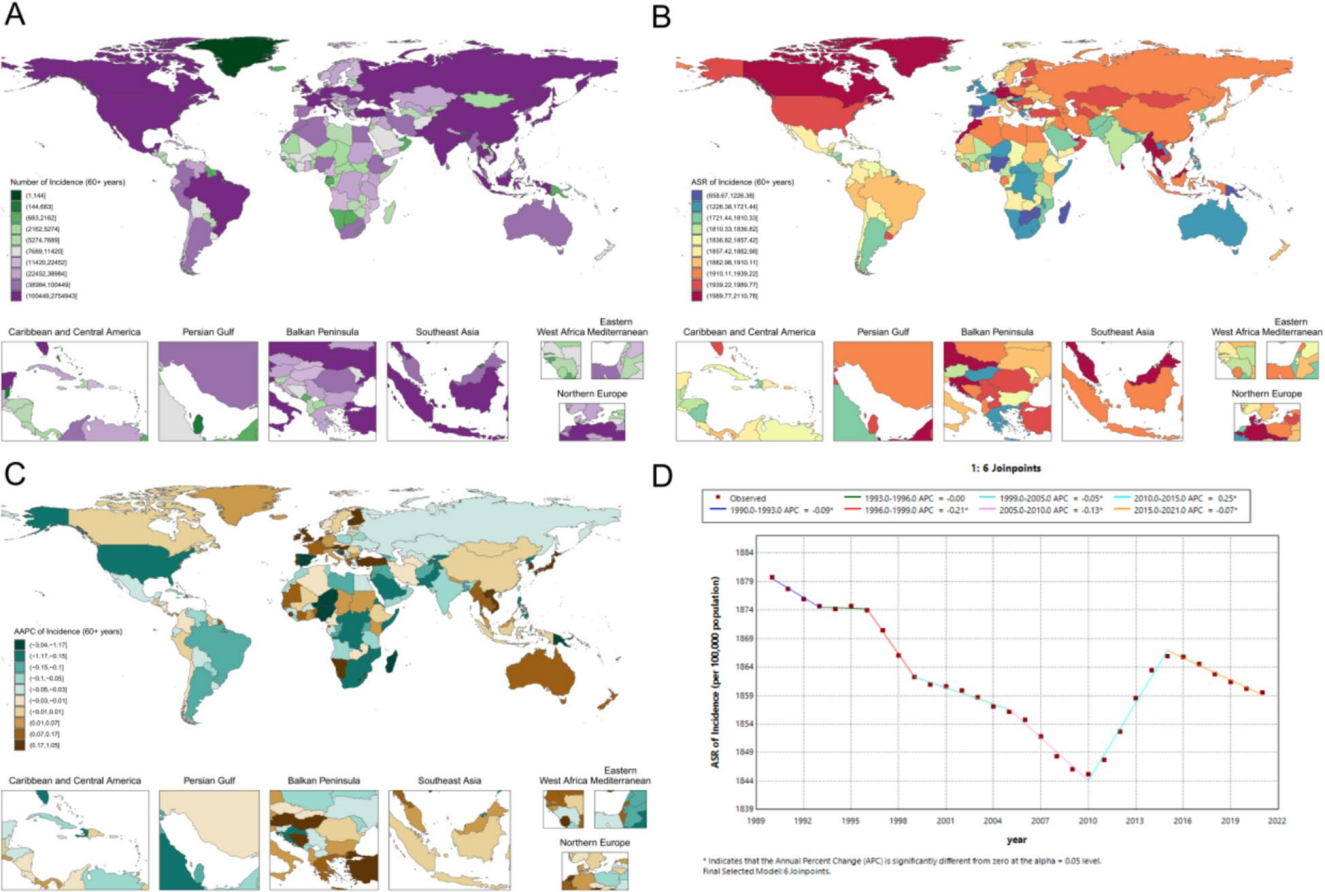
Older-age female periodontal disease metrics: **A** national case distribution in 2021; **B** national ASR distribution in 2021; **C** national AAPC of ASR, 1990–2021; **D** global Joinpoint analysis, 1990–2021

In 2021, the global ASR of periodontal disease among older women was 1859.5 (95% UI 1588.6–2110.8) per 100,000 population (Fig. 8A, B). The burden was particularly elevated in Southeast Asia, Central Asia, and several Middle Eastern countries: Malaysia (2057.9 [1058.0–2884.5] per 100,000), the Maldives (2038.1 [1000.9–2906.2] per 100,000), and Thailand (2110.8 [1215.5–2805.4] per 100,000), with Kazakhstan and Turkmenistan both exceeding 1960 per 100,000 and Qatar and the United Arab Emirates reaching 1989.7 (1075.8–2692.1) and 1992.1 (1085.8–2717.1) per 100,000, respectively. By contrast, several Pacific Island states and parts of sub-Saharan Africa exhibited relatively low ASRs, including Tuvalu at 826.0 (285.9–1504.7) per 100,000, Kiribati and the Solomon Islands at 740.8 (255.0–1369.6) and 749.4 (259.0–1353.1) per 100,000, respectively, and Niger and Burkina Faso both below 1300 per 100,000 (Fig. 8A, B). Among older women in East Asia, the ASR was 1936.0 (1300.5–2500.4) per 100,000 in China, 1901.3 (864.0–2808.6) per 100,000 in Japan, and 1736.6 (706.4–2751.0) per 100,000 in the Republic of Korea. Among high-income countries, Germany recorded an ASR of 2003.4 (1143.7–2681.1) per 100,000, while Norway and Switzerland reached 1876.2 (968.1–2612.0) and 1831.0 (796.3–2746.6) per 100,000, respectively; in contrast, Spain showed an unusually low level for a high-income country at 658.7 (219.0–1248.1) per 100,000 (Fig. 8A, B).

From 1990 to 2021, a six-joinpoint model (Fig. 8D) indicated periods of decline in 1996–1999 (APC − 0.21; p < 0.05) and 2005–2010 (APC − 0.13; p < 0.05), followed by a modest rebound during 2013–2019 (APC 0.11; p < 0.05). Regional temporal patterns varied (Fig. 8C): Vietnam showed a sustained increase (AAPC 0.79; p < 0.0001), Liberia a slight but significant decrease (AAPC − 0.05; p < 0.0001), China no significant long-term change (AAPC − 0.01; p = 0.5693), the Democratic People’s Republic of Korea a clear decline (AAPC − 0.28; p < 0.0001), and Turkey a marked increase (AAPC 0.44; p < 0.0001).

### Association between periodontal disease incidence and SDI

**Fig. 9:**
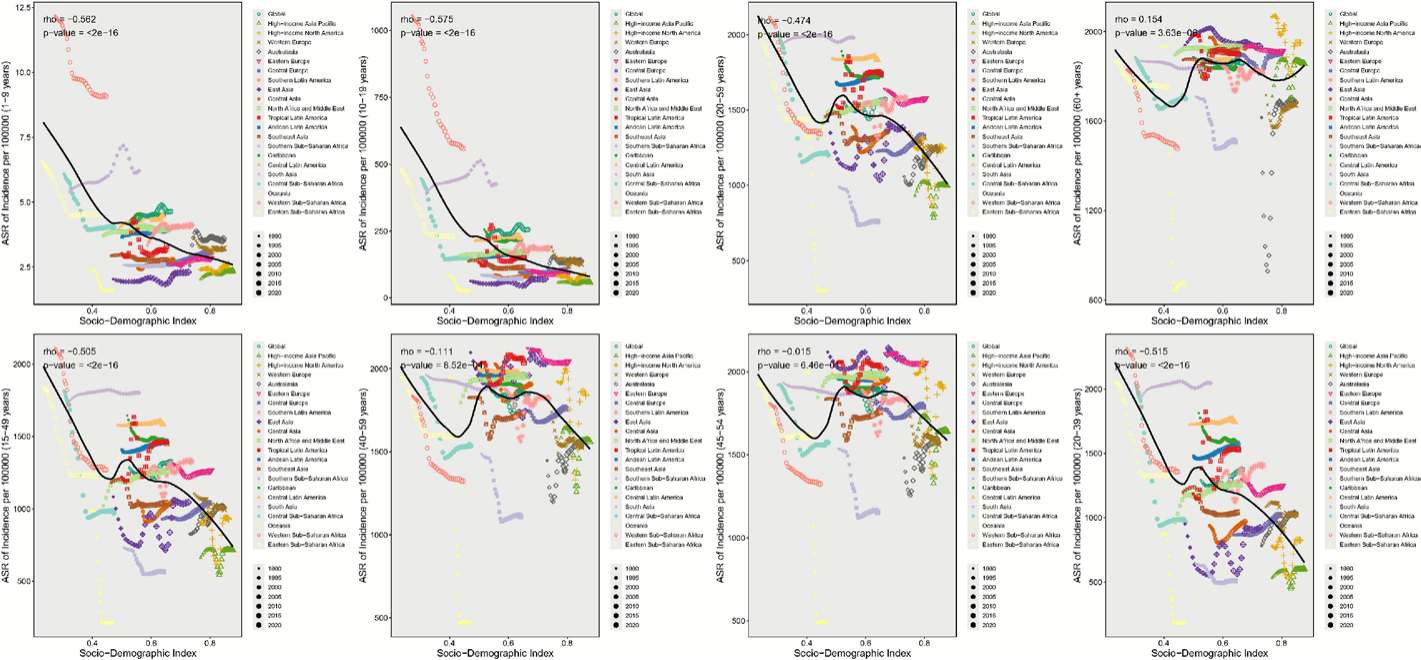
Relationship between Socio-demographic Index and age-standardised incidence of female periodontal disease in 21 GBD regions, 1990–2021

**Fig. 10:**
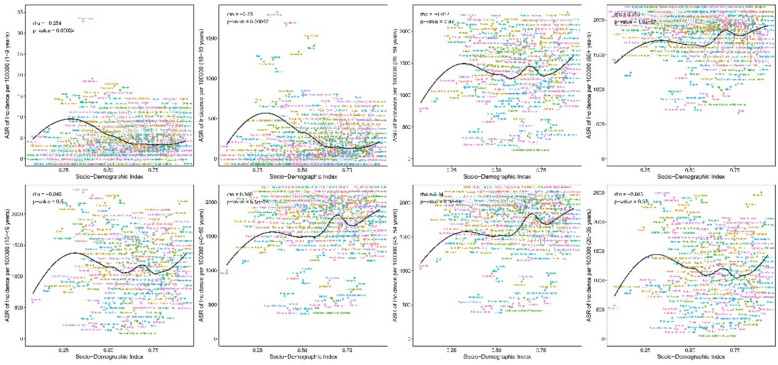
Relationship between Socio-demographic Index and age-standardised incidence of female periodontal disease in 204 countries and territories, 2021

In 2021, associations between ASR and SDI across life stages showed stage specificity and regional heterogeneity; during 1990–2021, associations between AAPC and SDI revealed development stage differences. At negatively correlated stages (higher SDI, lower ASR), for childhood (1–9 years), there was a significant negative correlation between ASR and SDI (rho = − 0.254, p = 0.00024): the ASR in low-SDI Sierra Leone was 32.82 per 100,000 population, 14 times higher than that in high-SDI China (2.31 per 100,000 population). A significant negative correlation between ASR and SDI was also observed in adolescence (10–19 years; rho = − 0.23, p = 0.00092), with Pakistan (820.2 per 100,000 population) and Sierra Leone (1823.8 per 100,000 population) identified as high-burden areas. Stages without significant association included adulthood and reproductive age: For adulthood (20–59 years), there was no significant correlation between ASR and SDI (rho = − 0.012, p = 0.87), with marked national differences—for example, the ASR was 2297.2 per 100,000 population in Denmark versus 467.5 per 100,000 population in Madagascar, with no linear association observed between the two. Among women of reproductive age (15–49 years), the correlation was weak and non-significant (rho = − 0.048, p = 0.50), which may reflect delayed childbearing in high-SDI regions (with elevated risk concentrated at ages 40–49) and underdiagnosis during pregnancy in low-SDI regions. These patterns suggest that analyses incorporating fertility behaviors, rather than SDI alone, are needed to better explain the observed association (Fig. 9). Positive correlations (higher SDI corresponding to higher ASR) mainly occurred in mid-to-late life stages: the correlation coefficient between ASR and SDI was rho = 0.306 (p = 8.5e–06) for the 40–59 age group and rho = 0.355 (p = 1.8e-07) for the 60 + age group. The ASR in high-SDI regions such as Canada was 2,077.6 per 100,000 population for the 40–59 age group, which exceeded that in low-SDI regions such as Nigeria (858.6 per 100,000 population), driven by population aging (60 + share > 20%), age-related increases in periodontal disease prevalence (> 70% affected > 65 years), and diagnostic rate differences (screening > 50% in high-SDI vs. < 15% in low-SDI), leading to underestimation in low-SDI regions. ASR and SDI showed dual thresholds: a low threshold SDI = 0.5, below which ASR declines rapidly with rising SDI (e.g., childhood ASR decreases > 40%); and a high threshold SDI = 0.8, above which ASR growth slows (e.g., 60 + ASR growth decelerates from 0.44%/year to 0.11%/year) (Fig. 10).

### Forecast of female periodontal disease ASR, 2022–2035

This section uses Autoregressive integrated moving average model (ARIMA) time-series models based on 1990–2021 ASR data by life stage to forecast global and key regional trends for 2022–2035. Forecast intervals (95% UI) are marked in figures and text, reflecting uncertainty.

**Fig. 11:**
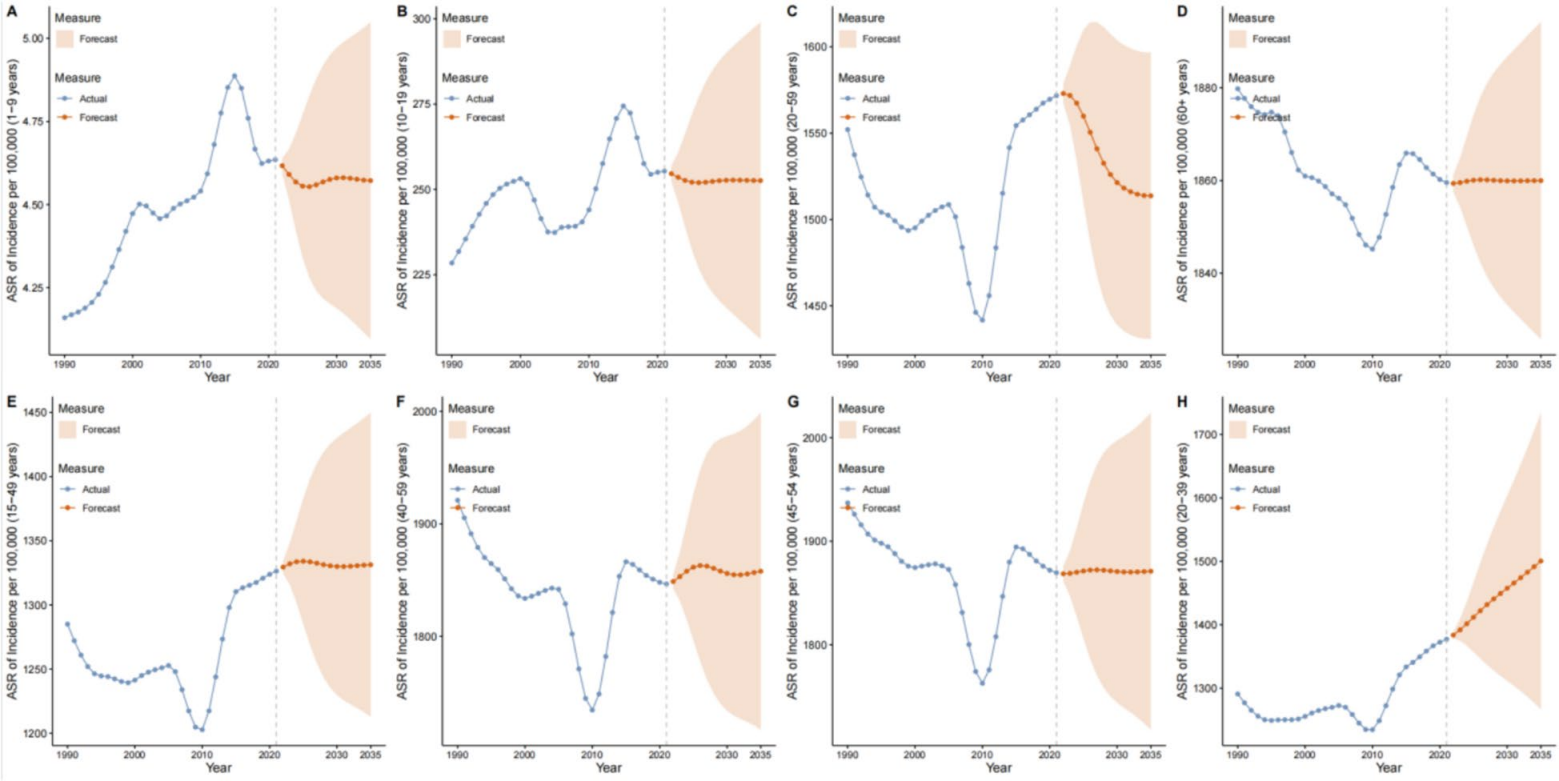
ASR trend projection

Childhood: From 2022 to 2035, the global female childhood ASR shows a stable, then slight decline pattern (Fig. 11A). ARIMA predicts 2022 ASR 4.62 (95% UI 4.59–4.64)/100,000, essentially unchanged from 2021 (4.64/100,000); 4.58 (95% UI 4.19–4.97) in 2030 and 4.57 (95% UI 4.10–5.05) in 2035. Forecast AAPC is about− 0.04, a notable slowdown from 1990–2021 (AAPC = 0.54).

Adolescence: From 2022 to 2035, global female adolescent ASR remains high and steady (Fig. 11B): 2022 ASR 254.63 (95% UI 252.40–256.86)/100,000 vs. 2021 (255.36/100,000); 252.70 (95% UI 215.41–289.99) in 2030; 252.59 (95% UI 206.16–299.02) in 2035. Fluctuations are < 1%, contrasting with the 1990–2021 increase (AAPC = 0.86), though differences remain between high-income countries and West Africa (e.g., 2035 U.S. 253.2/100,000 vs. Nigeria 251.8/100,000).

Reproductive age: From 2022 to 2035, the global age-standardized rate (ASR) of periodontal disease among women of reproductive age is projected to show a slow rise (Fig. 11E): the ASR will be 1329.43 (95% UI 1324.60–1334.26) per 100,000 population in 2022, representing a 0.23% increase compared with 2021 (1326.38 per 100,000 population); it will reach 1330.0 (95% UI 1229.87–1430.06) per 100,000 population in 2030 and 1331.33 (95% UI 1213.01–1449.64) per 100,000 population in 2035. The forecast annual growth rate (0.04%/year) is far lower than that recorded during 2010–2014 (APC = 2.14). Regionally, Sierra Leone will remain a high-burden area, with a projected ASR of 2401.5 per 100,000 population in 2035, while Kiribati will maintain a low ASR of 185.2 per 100,000 population.

Prime reproductive age: From 2022 to 2035, the global ASR of periodontal disease among women at prime reproductive age is expected to continue increasing but with slowing growth (Fig. 11H): the ASR will be 1383.63 (95% UI 1378.62–1388.65) per 100,000 population in 2022, rise to 1457.57 (95% UI 1311.62–1603.51) per 100,000 population in 2030, and reach 1500.50 (95% UI 1266.74–1734.26) per 100,000 population in 2035. The forecast average annual percentage change (AAPC) is approximately 0.71, which is lower than the 2010–2014 period (APC = 1.87). By country, the projected ASR in China will be 945.8 per 100,000 population in 2035, consistent with its historically stable trend (AAPC = -0.10); Denmark will maintain a high ASR of 2520.3 per 100,000 population with consistent growth.

Adulthood: From 2022 to 2035, global adult ASR shows stable, then slight decline (Fig. 11C): 2022 1572.99 (95% UI 1567.49–1578.48)/100,000, essentially unchanged from 2021; 1521.39 (95% UI 1439.02–1603.76) in 2030; 1513.75 (95% UI 1430.79–1596.71) in 2035. Forecast AAPC ~ − 0.32, reversing the fluctuating rise seen historically (2012–2018 APC = 0.41). Among high-income countries, Germany 2035 forecast 1980.5/100,000; Spain 665.3/100,000 (still low, smaller decline than historically).

Perimenopause: From 2022 to 2035, global perimenopausal ASR shows a slow rise (Fig. 11F): 2022 1848.75 (95% UI 1842.38–1855.13)/100,000; 2030 1855.93 (95% UI 1733.88–1977.99); 2035 1857.91 (95% UI 1716.97–1998.84). Forecast AAPC = 0.05, slower than the historical “fluctuating rise” (2005–2011 APC = 0.21). China 2035 forecast 1638.2/100,000 (consistent with historical stability, AAPC = − 0.10); DPRK 1530.5/100,000 (continuing historical decline with narrowing drop).

Menopause: From 2022 to 2035, menopausal ASR remains basically stable with minor fluctuations (Fig. 11G): 2022 1868.60 (95% UI 1861.55–1875.65)/100,000; 2030 1870.72 (95% UI 1744.17–1997.28); 2035 1871.04 (95% UI 1718.02–2024.05). Variability < 0.2%, consistent with the historical “no significant change” (e.g., China AAPC = − 0.09). By country, Cambodia 2035 forecast 1820.7/100,000 (continuing historical rise, AAPC = 0.42); Pakistan 2085.3/100,000 (continuing historical decline with reduced rate).

Older age: From 2022 to 2035, the ASR in older age remains stable (Fig. 11D): 2022 1859.38 (95% UI 1857.56–1861.20)/100,000; 2030 1859.92 (95% UI 1833.19–1886.65); 2035 1859.98 (95% UI 1825.81–1894.15). Variations < 0.04%; compared with the historical decline then rebound (2013–2019 APC = 0.11), the future enters a plateau. Among high-income countries, Spain 2035 forecast 662.1/100,000 (still low, consistent with history); Turkey 1890.5/100,000 (continuing historical rise, historical AAPC = 0.44, with slower growth).

## Discussion

Using data from the GBD 2021 study, this research stratified the female life course into eight stages based on physiological characteristics and systematically analyzed the disease burden, temporal trends, and association with the SDI of periodontal disease globally in women across 204 countries and territories from 1990 to 2021. The core findings complement existing studies and refine key dimensions: the ASR of periodontal disease in women globally exhibited significant life-course specificity—remaining consistently high from the prime reproductive age to older age, with the 2021 ASR reaching 1287.5 per 100,000 individuals for the prime reproductive age group and 1859.5 per 100,000 individuals for the older age group. The ASR showed a negative correlation with SDI in childhood and adolescence, a positive correlation in middle-to-late life, and a dual-threshold effect of SDI (ASR declined rapidly at an SDI of 0.5 and decelerated at an SDI of 0.8). This finding not only validates previous findings concerning the uneven global burden of periodontal disease [4, 28] but also reveals, for the first time, the interaction between physiological stages and socioeconomic development from the perspective of women’s full life course—providing more precise evidence for formulating gender-sensitive oral health policies.

This study demonstrated that the ASR of periodontal disease in postmenopausal women reached as high as 1582.6 per 100,000 in 2021, with a significant positive correlation with SDI (ρ = 0.355, p = 1.8e–07). This aligns with the results of Qi et al.’s [25] meta-analysis: decreases in estrogen levels in postmenopausal women exacerbate periodontal inflammation and bone resorption, and the global aging trend further intensifies disease management challenges for this demographic [26]. The ASR for reproductive-age women (15–49 years) was 1192.3 per 100,000, with a rapid increase during 2010–2014 (APC = 2.14, p < 0.05)—a trend closely associated with hormonal fluctuations during pregnancy and lactation that contribute to exacerbated gingival inflammation [8]. Notably, this study included a newly defined subgroup of “prime reproductive age (20–39 years)” and found that the ASR for this subgroup (1287.5 per 100,000) was higher than that observed in the broader reproductive age group, with sustained growth still observed during 2014–2021 (APC = 0.62, p < 0.05). This refined result addresses a key limitation of Dai et al.’s [27] study, which solely focused on the overall trend of the reproductive age group—highlighting the need for more targeted interventions tailored to women in their peak childbearing years.

From a regional perspective, this study confirmed that low-to-middle Socio-Demographic Index (SDI) regions are high-burden areas for periodontal disease in women: in 2021, the age-standardized rate (ASR) of reproductive-age women in Sierra Leone (West Africa) reached 2348.2 per 100,000, and that in Gambia was 2294.4 per 100,000—both significantly higher than the global average. This aligns with the findings of Zhang et al. [28], who reported that the burden of periodontal disease is concentrated in low-resource regions. However, this study further identified that the burden among middle-to-late-aged women is prominent in high-SDI regions (e.g., the ASR of postmenopausal women in Denmark was 2145.7 per 100,000, and that of older women in Germany was 2003.4 per 100,000). These drivers differ fundamentally from those in low-to-middle SDI regions: the high burden in high-SDI regions stems from population aging (the proportion of individuals aged ≥ 60 years exceeds 20%) and improved diagnostic rates (screening coverage > 50%) [29], whereas the high burden in low-to-middle SDI regions is primarily driven by insufficient oral health resources (e.g., the overburdened healthcare system in Gambia [30]), poor sanitation, and underdiagnosis (screening coverage < 15%) [31].

Socioeconomic and cultural factors significantly shape the distribution of disease burden. Specifically, economic development can reduce periodontal disease incidence by improving access to dental care and fluoride toothpaste, while improved sanitation, such as through clean water supplies, and greater oral health awareness, which promotes regular toothbrushing, are also protective factors [28]. However, in regions such as Pakistan [32] and Mediterranean countries [33], high dietary carbohydrate intake and inadequate oral hygiene practices, including infrequent brushing and low dental visit frequency, offset part of the benefits derived from development. Educational level is negatively correlated with the risk of periodontal disease. A relationship supported by studies conducted in Qatar [34] and the Lisbon Metropolitan Area [35]. And the present study further confirmed that this negative association is more pronounced in reproductive-age and perimenopausal women. In addition, racial disparities cannot be overlooked: Susin et al. [36] found that aggressive periodontitis is most prevalent in African and African-descent populations, a finding consistent with the high burden of periodontal disease observed in sub-Saharan Africa in this study. Thus reflecting the interplay of genetic background, living environment, and hygiene practices [37].

From 1990 to 2021, the global age-standardized rate (ASR) of periodontal disease in women exhibited stage-specific increases: steady rises in childhood (AAPC = 0.54) and adolescence (AAPC = 0.86), with a peak increase in the reproductive age group during 2010–2014 (APC = 2.14, p < 0.05). This is closely linked to global aging, lifestyle shifts (e.g., high sugar consumption), and inequitable allocation of oral health resources [27, 28]. However, trend heterogeneity was observed across stages; for instance, the ASR of adolescents in West Africa showed a declining trend (AAPC = -0.30, p < 0.001), indicating that adolescent oral health interventions in some regions have achieved initial efficacy and providing replicable insights for other regions (Figs. 2D, 3D). Based on the study findings, targeted policy recommendations are as follows: (1) Low-to-middle Socio-Demographic Index (SDI) regions should increase investment in oral health for children and adolescents, and promote school-based oral screening and fluoride application (e.g., Finland reduced the periodontal disease prevalence among 15-year-olds from 32 to 18% via its “Adolescent Oral Health Program”); (2) High-SDI regions should strengthen periodontal health management for middle-to-late-aged women and integrate periodontal screening into postmenopausal health check-ups (e.g., Japan included periodontal examinations for women aged ≥ 60 years in routine medical insurance coverage); (3) All regions should prioritize pre-pregnancy oral care for reproductive-age women and incorporate it into maternal and child health packages (e.g., Thailand made pre-pregnancy periodontal screening a mandatory item in prenatal care, reducing the risk of periodontal disease-associated adverse pregnancy outcomes by 27%). These measures have been validated as effective in select countries and are feasible for large-scale scaling-up.

This study has several limitations: Given that this analysis draws on GBD data, it relies on model-derived estimates, which may be influenced by the accuracy of raw data and modeling assumptions. It did not differentiate between periodontitis and gingivitis, potentially leading to underestimation of the burden of mild lesions. This is particularly impactful for the accuracy of assessments in adolescent and reproductive-age women. Although the stratification of the female life course into stages was based on physiological characteristics and the GBD framework. It requires further validation using large-scale clinical data, especially for defining periodontal disease changes during perimenopause. In addition, disease burden may be underestimated in low-income countries due to limited screening capacity and incomplete medical records [29].

Future research should focus on three areas: (1) Validating the physiological mechanisms underlying periodontal disease across different life course stages using cohort studies (e.g., the association between hormone levels and periodontal inflammatory markers); (2) Assessing the cost-effectiveness of oral health interventions, particularly targeted interventions for women in the prime reproductive age and perimenopausal stages; (3) Expanding the data scope to incorporate individual-level factors (e.g., diet, oral hygiene practices) to further elucidate the interaction between socioeconomic status and physiological characteristics.

## Conclusion

The female burden of periodontal disease has life-course dynamics and regional differences, related to physiological and socioeconomic factors. Disease risk varies systematically with life stage: reproductive age, perimenopause, and older age carry heavier burdens; childhood and adolescence have lower incidence but large regional disparities due to resource imbalance. Globally, risk is higher among youth in low-SDI regions, prominent in middle-older ages in high-SDI regions, and balanced in middle-SDI regions. However, the availability of oral health services often does not match these shifting needs, leading to mismatches between service supply and disease burden during transitions between life stages. From 1990 to 2021, risks in younger ages rose slightly, then stabilized after midlife; high-SDI regions rose slightly; peaks in reproductive age point to pregnancy-focused interventions. These findings underscore the importance of stage-tailored interventions aligned with physiological changes, remediation of resource gaps according to SDI level, and adjusted management priorities to improve women’s periodontal health globally.

## Supplementary Information

Supplementary Material 1. Supplementary Figure S1. Global distribution of Socio-demographic Index (SDI) levels across 204 countries and territories, 2021.

## Data Availability

The data used in this study are available free of charge online at https://vizhub.healthdata.org/gbd-results on request. The datasets used and/or analysed during the current study available from the corresponding author on reasonable request.
