# Supplementary figures and images for: Exploring the age-dependent burden of periodontal disease in women across different socio-demographic levels
Source: Trop Med Health. 2025 Dec 26;54:14. doi: 10.1186/s41182-025-00886-3 (PMC12805735; doi:10.1186/s41182-025-00886-3)

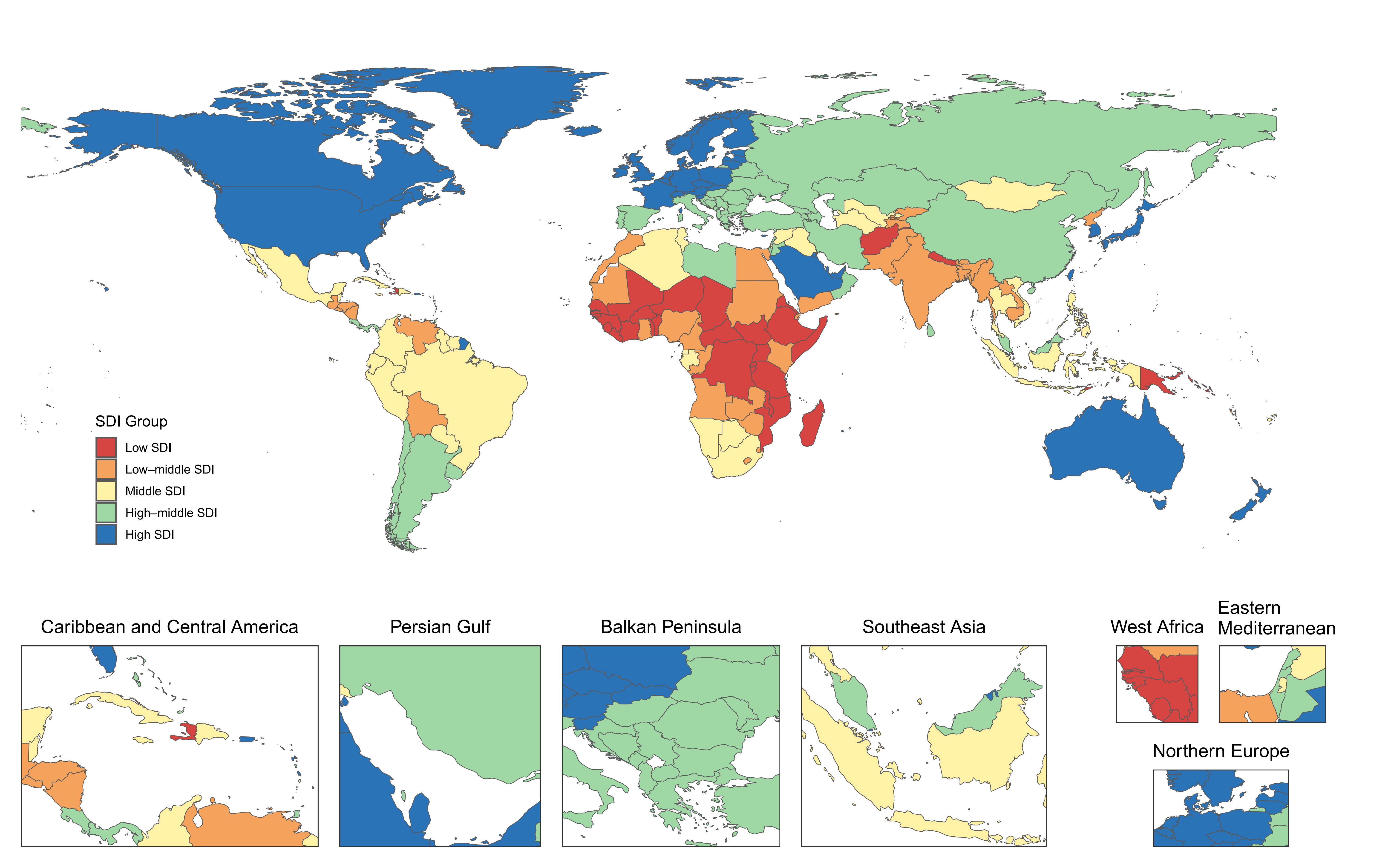

Supplement: Supplementary file 1 — Supplementary Material 1. Supplementary Figure S1. Global distribution of Socio-demographic Index (SDI) levels across 204 countries and territories, 2021. [file 41182_2025_886_MOESM1_ESM.png]
